# Binocular Viewing Facilitates Size Constancy for Grasping and Manual Estimation

**DOI:** 10.3390/vision6020023

**Published:** 2022-04-20

**Authors:** Ewa Niechwiej-Szwedo, Michael Cao, Michael Barnett-Cowan

**Affiliations:** Department of Kinesiology and Health Sciences, University of Waterloo, Waterloo, ON N2L 3G1, Canada; michael.cao.22@gmail.com (M.C.); mbc@uwaterloo.ca (M.B.-C.)

**Keywords:** binocular, grasping, grip aperture, monocular, size constancy

## Abstract

A prerequisite for efficient prehension is the ability to estimate an object’s distance and size. While most studies demonstrate that binocular viewing is associated with a more efficient grasp programming and execution compared to monocular viewing, the factors contributing to this advantage are not fully understood. Here, we examined how binocular vision facilitates grasp scaling using two tasks: prehension and manual size estimation. Participants (*n* = 30) were asked to either reach and grasp an object or to provide an estimate of an object’s size using their thumb and index finger. The objects were cylinders with a diameter of 0.5, 1.0, or 1.5 cm placed at three distances along the midline (40, 42, or 44 cm). Results from a linear regression analysis relating grip aperture to object size revealed that grip scaling during monocular viewing was reduced similarly for both grasping and estimation tasks. Additional analysis revealed that participants adopted a larger safety margin for grasping during monocular compared to binocular viewing, suggesting that monocular depth cues do not provide sufficient information about an object’s properties, which consequently leads to a less efficient grasp execution.

## 1. Introduction

Veridical representation of object size and distance is critical for perceiving and acting in our three-dimensional world. A particular object’s retinal image size varies with distance; however, the perception of its size is maintained over a large range of distances, which is referred to as size constancy. Similarly, binocular retinal disparity of a particular object’s extent in depth varies with distance, but depth constancy ensures that the object’s perceived depth remains consistent [1]. Size constancy is maintained by scaling the object’s retinal size with the estimate of the object’s distance. Conversely, depth constancy is maintained by scaling retinal disparity with the square of the distance. Studies show that distance estimation relies on multiple monocular and binocular depth cues which are weighted based on their reliability [2]. Moreover, the contribution of these cues varies depending on the task, context, and previous experience [3,4,5,6,7]. For example, it has been suggested that binocular depth cues are particularly important for action tasks, such as grasping when compared to perceptual size estimation tasks [8,9]. Here we examine how binocular viewing facilitates size constancy during grasping and manual size estimation.

Seminal neuroanatomical and behavioural studies demonstrated that action and perception engage distinct neural networks emerging from the primary visual cortex [10,11]. The dorsal pathway projects to multiple regions of the parietal cortex involved in action planning and execution, while the ventral pathway projects to occipitotemporal areas involved in object recognition. The neural mechanisms underlying size constancy have been investigated using brain imaging. Results reveal that activation in the primary visual cortex reflects the perceived rather than the retinal object size, indicating that neural activity at early stages of visual processing reflects size constancy [12,13]. Notably, lesions affecting the primary visual cortex and ventral stream areas disrupt size constancy for perceptual estimates of object size but allow reliable scaling of grip aperture during grasp execution [14]. This suggests that different neural substrates may support size constancy mechanisms for perceptual judgments and motor tasks which may engage the ventral and dorsal networks differentially [15]. Notably, the strict dissociation between the visual streams has been questioned [16,17,18] while the nature of the interaction between dorsal and ventral streams remains an area of active exploration [19,20,21,22].

Distance estimation relies on multiple monocular cues including texture gradients, height in the visual field, linear perspective, occlusion, motion parallax, blur or accommodation, as well as binocular cues including retinal disparity and ocular vergence. Size constancy could be achieved using any combination of these cues. Indeed, when all depth cues are available, size constancy is comparable for grasping and perceptual tasks, as reflected by the slope relating grip aperture and object size [23]. The role of binocular cues in prehension has been studied extensively using various experimental manipulations and distance cues [24,25,26,27,28,29]. Size constancy is disrupted significantly under severely restricted conditions such as viewing objects monocularly via a pinhole in a dark environment [23]. However, studies with less restrictive manipulations using multiple monocular cues also reported a larger grip aperture and poorer grasp execution [27,30]. It is not known to what extent these deficits could be attributed to a reduced ability to scale grip aperture with object size. Thus, the current study assessed the role of binocular cues in grip aperture scaling during grasping in a fully lit room where other monocular cues were not restricted, and performance on the action task was compared to a perceptual size estimation task.

Binocular viewing provides two unique depth cues: retinal disparity and ocular vergence. Horizontal retinal disparity is the basis of stereopsis, which provides a vivid impression of depth and is a potent cue for an object’s 3D structure [31]. Conversely, ocular vergence provides metric information about egocentric distance and contributes to maintaining depth constancy [1]. Both stereopsis and ocular vergence provide reliable distance cues in the peripersonal space; however, their reliability is reduced for distances beyond two meters [32]. Reducing stereopsis in visually normal adults disrupts grasp execution [33,34]. Likewise, patients with abnormal stereopsis have deficits in grasping, in particular larger grip aperture and prolonged grasp duration [35]. Thus, binocular disparity provides important input for efficient grasp execution. Indeed, monkey neurophysiology and human neuroimaging studies have found disparity in sensitive neurons and neural activity in various areas along the dorsal stream, specifically area AIP, which is involved in grasp planning [36,37,38]. Recent studies have also revealed disparity-related activity in the ventral stream, which may have an important role in object recognition [39,40].

The contribution of binocular viewing to the performance of grasping and manual estimation was assessed in a patient with visual agnosia due to a lesion mainly affecting the ventral areas (patient DF). Despite significant deficits in manual estimation of object size, scaling of grip aperture with object size was accurate when grasping during binocular viewing [8,41,42]. Notably, grasping performance was significantly reduced when the same action was performed during monocular viewing, suggesting that binocular cues provide unique input for grasp execution. Support for the importance of binocular cues for grip aperture scaling in neurologically intact participants is mixed. One study used the Ebbinghaus stimulus and showed that grasping under monocular viewing was susceptible to the illusion, which supports binocular cues as being important for accurate grip scaling [9]. In contrast, a study which examined grip scaling using Muller-Lyer illusion stimuli during perceptual matching and grasping tasks found no significant difference between binocular and monocular viewing [43]. Perhaps such findings are not surprising in light of other studies showing that illusions may have a similar effect on perception and action [44]. Therefore, it is important to re-examine the role of binocular depth cues during the performance of tasks that do not rely on an illusion.

To summarize, grasping decrements during monocular compared to binocular viewing are well documented in studies assessing prehension using cylinders or blocks and instructions that emphasize speed and accuracy [7,24,27,29]. The main finding from these studies is increased grip aperture and less efficient grasp execution during monocular viewing. However, these studies did not examine grip aperture scaling, thus, the question remains to what extent is the documented grasping deficit explained by reduced size constancy when binocular cues are missing? Size constancy or the accuracy (responsiveness) of grip scaling is reflected by the slope relating grip aperture and object size, where a slope of one indicates veridical scaling. Grip aperture scaling with object size has been well documented for grasping during binocular viewing; see review by [45] with an average slope value of 0.82. In contrast, slope values range between 0.90 to 1.85 for a manual estimation task [14,46,47,48]. To our knowledge, the large difference in grip scaling responsiveness between action and manual estimation has not been fully explained in the literature.

If binocular depth cues provide important input for programming grip aperture, and thus contribute to accurate grip scaling, we hypothesize that size constancy will be reduced during monocular viewing. Moreover, it is possible that binocular depth cues are used preferentially for action compared to perception; thus, monocular cues could provide sufficient input for manual estimation. Accordingly, we expected that size constancy will be reduced to a greater extent during monocular viewing for grasping in comparison to manual estimation. Alternatively, it is possible that grip aperture scaling is maintained during monocular viewing and the increased aperture during grasping reflects a larger safety margin, which is a tendency to adopt a larger grip opening to ensure successful contact with the object [49,50]. Consistent with previous work on size constancy, these hypotheses were evaluated using a linear regression relating object size and grip aperture where the slope reflects the responsiveness (i.e., accuracy) of grip scaling, which is a proxy measure of size constancy. Here we propose that the intercept value reflects the safety margin for grasping. In general, a higher safety margin is adopted when the uncertainty of sensory input increases due to reduced visual feedback [51,52] or when motor variability increases for faster movements [53].

## 2. Materials and Methods

### 2.1. Participants

Thirty visually healthy participants (16 females, 14 males, mean age 21.3 years, SD = 2.3) were recruited. Two participants were excluded due to poor data quality resulting from missing markers. Participants had visual acuity 20/20 or better in each eye (Bailey–Lovie’s vision chart), and a stereoacuity of 40 s of arc or better (Preschool Randot Stereo test). All participants were right hand dominant based on self-report, and twenty-six were right eye dominant, which was assessed by the Miles test [54]. Participants were asked to extend their arms and to look through an opening formed by joining their hands. They were asked to fixate on an object through the opening, and then alternately close one eye and then the other, and to report which eye was still fixated on the object—this was determined as the dominant eye. This work was carried out in accordance with the 2008 Code of Ethics of the World Medical Association (Declaration of Helsinki). The study’s protocol was approved by the University of Waterloo Research Ethics Committee (ORE#21497). Participants signed a written consent prior to participating.

### 2.2. Experimental Design

**Figure 1:**
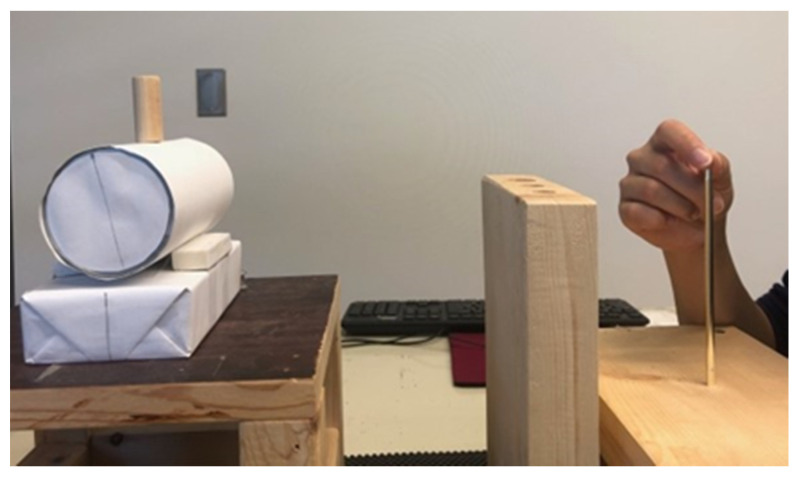
Experimental setup. Participants were seated in front of the apparatus in a fully lit environment. For the grasping task, they were asked to reach, grasp, and transport the cylinder as fast as possible. For the manual estimation task, they were asked to open the thumb and index finger to indicate the size of the cylinder.

The experimental setup is illustrated in Figure 1. Participants were seated with their head supported by a chin rest in front of the apparatus. They performed a prehension task and a manual estimation task during binocular and monocular viewing, which were randomized in blocks across participants. Monocular viewing was implemented by covering the non-dominant eye with a translucent eye patch. Upper limb kinematics were recorded using a motion capture system (Optotrak 3D Investigator, NDI, Waterloo, ON, Canada) at a sampling frequency of 500 Hz. The system was calibrated using a standardized calibration probe to define the coordinate system as follows: *x*-axis (azimuth), *y*-axis (elevation), *z*-axis (depth). Two small infrared markers (7 mm diameter) were attached using tape on the distal phalanges of the index finger and thumb of the dominant hand.

Each trial began with participants’ eyes closed, and their index and thumb in a standardized position holding the tip of a blunt needle located 10 cm in front of the trunk’s midline. A cylindrical object (height: 30 mm; diameter: 5 mm, 10 mm, and 15 mm) was placed at 40 cm, 42 cm, or 44 cm directly in front of the start position at approximately eye level. Object size and distance were randomized so that participants had to plan their movement on each trial. The cylinder was placed on a curved surface, which increased the demand for limb accuracy and precision. The prehension task involved reaching, grasping, and transporting the cylinder to a platform located 25 cm from the trunk to fit the cylinder into one of the three slots which matched the cylinder’s diameter. A verbal cue was used as a ‘Go’ signal, and participants were instructed to open their eyes and complete the prehension task as fast as possible while maintaining accuracy. For the manual estimation task participants were instructed to look at the cylinder and estimate its size using the thumb and index finger, and to hold that position for 2 s. Vision of the limb was not restricted during the prehension or estimation tasks. There were 10 trials in each experimental condition where object size, distance and task were randomly interleaved.

### 2.3. Data Reduction

A custom Matlab script (Matlab, Mathworks, Natick, MA, USA) was used to analyze limb kinematic data. First, a low pass, second order Butterworth filter with a cut-off frequency of 10 Hz was applied to the raw position data. All trials were screened visually by one of the authors. Grip aperture was calculated as the difference of the vector between the finger and thumb marker. For the prehension task, maximal grip aperture (MGA) was defined as the largest distance between the finger and thumb marker during the reaching movement. For the manual estimation task, grip aperture was also calculated as the vector between the finger and thumb markers once the velocity of the markers fell <20 mm/s and remained there for 1 s.

Additional kinematic measures were calculated as follows. Peak velocity was defined as the maximum velocity along the depth direction for reaching towards the cylinder. Total movement duration was defined as time from reach initiation (index finger velocity exceeded 30 mm/s for 20 ms) to time of reach termination (index finger velocity dropped to 100 mm/s). Movement duration was further portioned into the acceleration interval (time from reach initiation when index finger velocity exceeded 30 mm/s for 20 ms to time of peak velocity) and deceleration interval (time from peak velocity to time of reach termination when the index finger velocity dropped to 100 mm/s). Grasp duration was calculated based on the velocity of the finger and thumb. Specifically, grasp initiation was defined as the time when finger velocity in the *z*-axis fell under 100 mm/s for 20 consecutive milliseconds after peak velocity, and grasp termination was defined as the time when thumb velocity exceeded at least 30 mm/s for 20 consecutive milliseconds, which indicated that the cylinder was being transported to the next location. These criteria are consistent with the aiming literature [55,56,57], and our previous work on prehension [7,30].

### 2.4. Statistical Analysis

**Figure 2:**
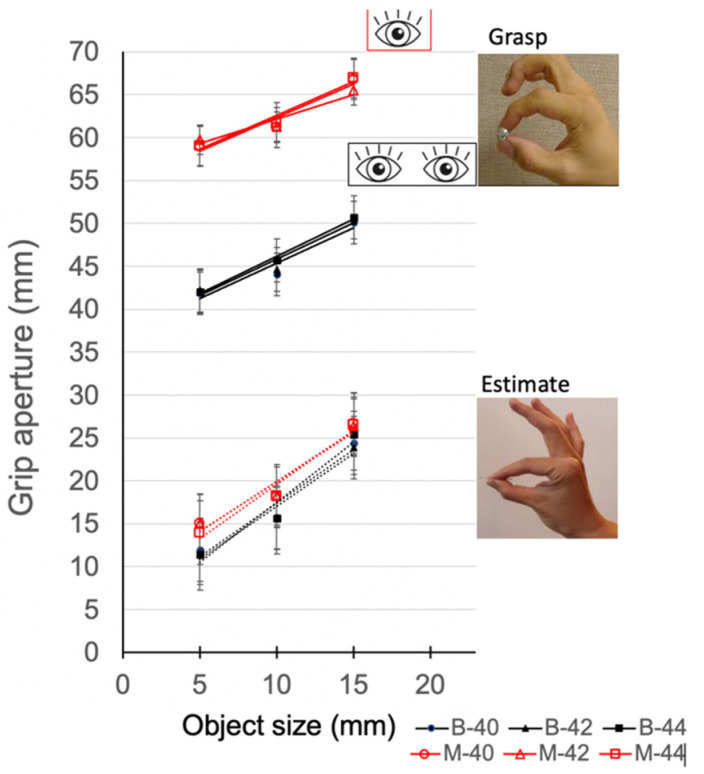
Mean grip aperture plotted for the grasping task (solid lines) and estimation task (dashed lines). Error bars show ±1 standard error. B: binocular (depicted by black symbols); M: monocular (depicted by red symbols); B40/M40 objects presented at 40 cm; B42/M42: objects presented at 42 cm; B44/M44: objects presented at 44 cm.

Statistical analyses were conducted using the Statistical Analysis System (SAS) Studio, ver. 3.5 Enterprise Edition (SAS Institute Inc., Cary, NC, USA). Univariate analysis was used to confirm normality of outcome measures. Mean grip aperture was calculated across the experimental conditions for each participant. As shown in Figure 2 and confirmed by statistical analysis, mean grip aperture was comparable across the distances tested for a given viewing condition and object size. Thus, the results were collapsed, and distance was not considered in further analysis of grip aperture. Data were submitted to a 3-way repeated measures analysis of variance (ANOVA) to assess the effect of task (grasping, estimation), viewing condition (binocular, monocular), and size (5, 10, 15 mm) on the mean and precision (i.e., standard deviation of grip aperture) of grip aperture.

Size constancy was assessed using a linear regression analysis relating object size and grip aperture for each experimental condition across participants. Here, the slope reflects the responsiveness of grip aperture with changing object size while the intercept could be interpreted as a safety margin, such that a greater grip aperture indicates a larger safety margin. The slope and the intercept values were analyzed using a repeated-measures ANOVA with two factors: task (grasping, estimation) and viewing condition (binocular, monocular).

Reach-related measures such as reach duration, peak velocity, duration of acceleration and deceleration intervals, and grasp duration were analyzed using repeated measures ANOVA with 3 factors: viewing condition (binocular, monocular) distance (40, 42, 44 cm), object size (5, 10, 15 mm). A linear regression analysis relating peak velocity with distance was used to calculate the slope which reflects scaling of reach peak velocity with distance, and the intercept which reflects the extent of caution and safety margin in reach execution. We propose that lower intercept (i.e., lower reach velocity) indicates increased caution and a greater safety margin. The slope and the intercept values were submitted to a repeated-measures ANOVA with one factor: viewing condition (binocular, monocular). Significant effects were deconstructed by conducting post-hoc tests using Tukey’s HSD test, and significance level was set at *p* < 0.05.

Finally, analysis was conducted to determine whether the safety margin for reaching and grasping are related. A Pearson’s correlation analysis using the intercept from the linear regression relating grip aperture and size, and peak velocity and distance, was performed for each viewing condition.

## 3. Results

### 3.1. Mean Grip Aperture

Figure 2 shows the mean grip aperture across all experimental conditions. As illustrated in the figure, and confirmed by the results from a 4-way ANOVA, object distance was not a significant factor (F_(2,54)_ = 2.79, *p* = 0.059) indicating that mean grip aperture was scaled similarly across the distances for the three objects used in this study. Grip aperture was larger during grasping compared to the estimation task (F_(1,27)_ = 20.13, *p* < 0.0001), during monocular vs. binocular viewing (F_(1,27)_ = 127.83, *p* < 0.0001), and as object size increased (F_(2,54)_ = 337.46, *p* < 0.0001). A significant task by viewing condition interaction (F_(1,27)_ = 124.27, *p* < 0.0001) revealed that grip aperture was significantly larger during grasping while viewing monocularly compared to the binocular condition with a mean difference of 16.8 mm (*p* < 0.0001). In contrast, the mean difference between viewing conditions for the estimation task was 2.6 mm (*p* = 0.012).

### 3.2. Precision Grip Aperture

**Figure 3:**
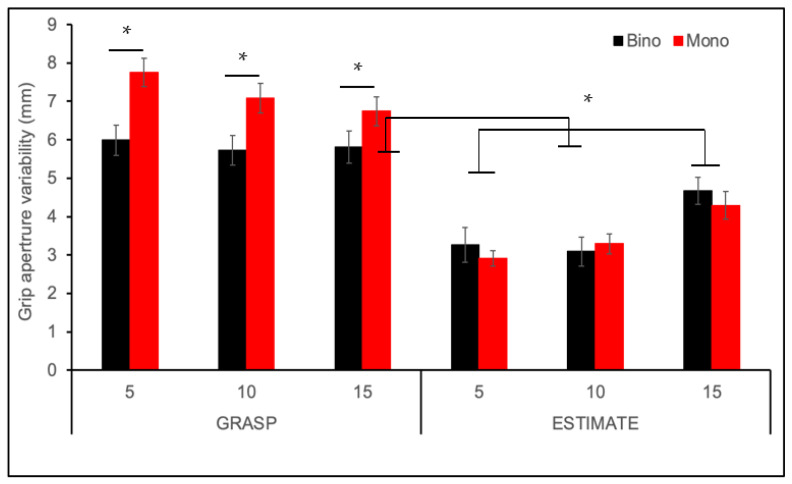
Grip aperture variability for binocular (Bino) versus monocular (Mono) viewing plotted across all experimental conditions. Error bars show ±1 standard error. Grip aperture was significantly more variable during grasping compared to estimation, as well as during monocular binocular viewing during grasping (* *p* < 0.05). There was no effect of viewing condition for the estimation task.

Grip aperture precision was assessed by calculating the standard deviation of grip aperture, which is plotted in Figure 3. Higher grip aperture variability means that precision is lower. Grip aperture variability was higher during grasping compared to the estimation task (F_(1,27)_ = 96.02, *p* < 0.0001), and during monocular compared to binocular viewing (F_(1,27)_ = 5.08, *p* = 0.033). The effect of viewing condition was driven by a large increase in grip aperture variability in the grasping task, confirmed by 2-way interaction between task and viewing condition (F_(1,27)_ = 10.11, *p* = 0.004). Post-hoc testing revealed that variability was higher during monocular compared to binocular viewing for the grasping task (*p* = 0.005). In contrast, grip aperture precision was similar across viewing conditions for the estimation task (*p* = 0.608). There was also a significant 3-way interaction (F_(6,162)_ = 8.33, *p* < 0.0001). Post-hoc testing indicated that for the estimation task grip aperture was more variable for the largest object size in both viewing conditions (*p* < 0.0001), which is consistent with Weber’s law [58]. In contrast, during grasping, grip aperture was slightly but not significantly less variable for the larger object size (*p* = 0.549).

### 3.3. Slope and Intercept Analysis for Grip Aperture

**Figure 4:**
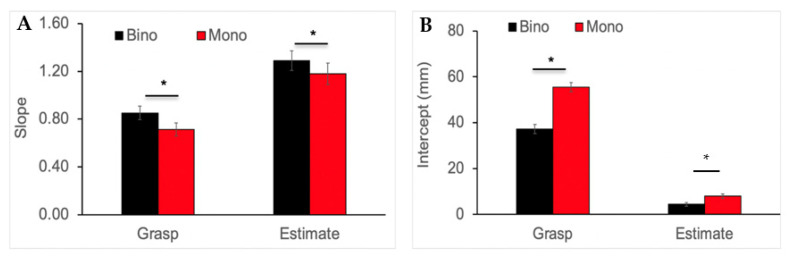
Mean slope (**A**) and intercept (**B**) values from a regression analysis relating grip aperture and object size for grasping and estimation tasks across the viewing conditions. Error bars show ±1 standard error. The slope was significantly lower, and the intercept was significantly higher for the grasping in comparison to the estimation task. Monocular viewing was associated with lower slope and a higher intercept for both tasks (* *p* < 0.05).

The mean slopes obtained from the regression analysis relating grip aperture and object size are shown in Figure 4A. As expected, the slope was significantly higher for the manual estimation task compared to the grasping task (F_(1,27)_ = 25.90, *p* < 0.0001). Results also showed a main effect of viewing condition F_(1,27)_ = 7.84, *p* = 0.009). In contrast to our hypothesis, the interaction was not significant (F_(1,27)_ = 0.15, *p* = 0.699), indicating that removing binocular depth cues was associated with a reduction in grip aperture responsiveness; however, the magnitude of this effect was similar for the grasping and estimation task.

Figure 4B displays the mean intercept results from the regression analysis. All effects were significant: task (F_(1,27)_ = 546.31, *p* < 0.0001), viewing condition (F_(1,27)_ = 109.96, *p* < 0.0001), and interaction (F_(1,27)_ = 116.99, *p* < 0.0001). As illustrated in the figure, the intercept was significantly higher during grasping compared to the estimation task (*p* < 0.0001), which was further exacerbated during monocular viewing (*p* < 0.0001). The intercept value was also higher during monocular compared to binocular viewing for the estimation task (*p* = 0.011); however, the significant interaction indicates that the difference for the grasping task was relatively greater.

### 3.4. Reach Kinematics

**Figure 5:**
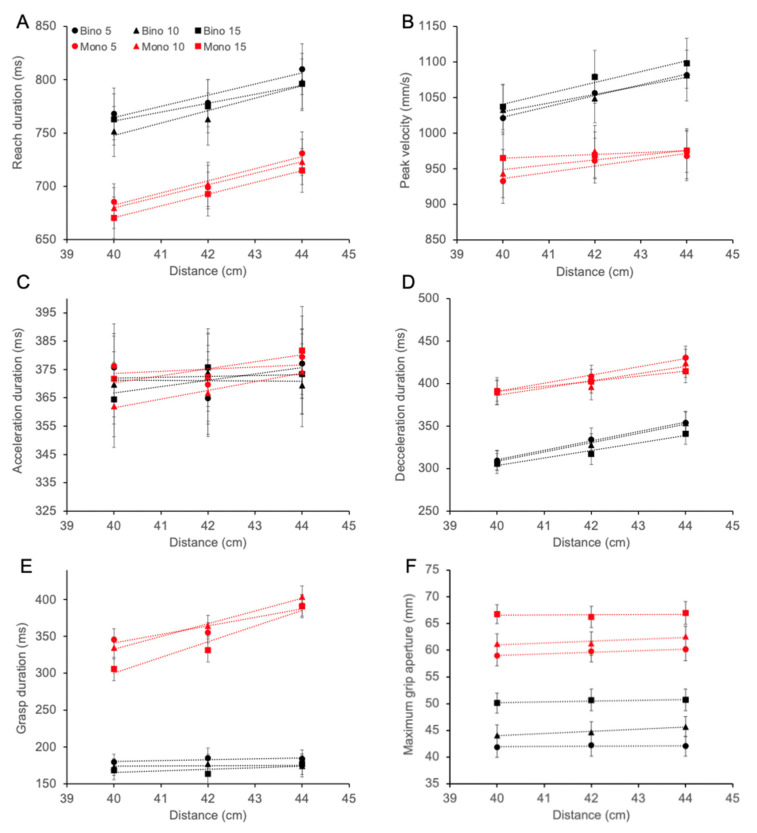
Mean kinematics obtained at the 3 viewing distances during binocular (black) and monocular (red) viewing: reach duration (**A**); peak velocity (**B**); acceleration duration (**C**); deceleration duration (**D**); grasp duration (**E**); maximum grip aperture (**F**). The lines are fitted using a linear regression. Error bars show ±1 standard error. Different symbols indicate object’s size (circle: 5 mm cylinder; diamond: 10 mm cylinder; square: 15 mm cylinder).

Results showing the effects of viewing condition, distance and size for all kinematic measures are depicted in Figure 5. As expected, peak velocity was higher (F_(2,54)_ = 36.85, *p* < 0.0001) and reach duration (F_(2,54)_ = 55.45, *p* < 0.0001) and deceleration interval (F_(2,54)_ = 65.70, *p* < 0.0001) were longer as distance increased. The reaching movement was performed slower (F_(1,27)_ = 40.89, *p* < 0.0001), with lower peak velocity (F_(1,27)_ = 46.93, *p* < 0.0001) and a longer deceleration interval (F_(1,27)_ = 78.60, *p* < 0.0001) during monocular viewing. No significant effects were found for the duration of acceleration interval. Unexpectedly, a main effect of object size was found for reach duration (F_(2,54)_ = 3.78, *p* = 0.029; however, post-hoc testing adjusted for multiple comparisons did not reveal differences), peak velocity (F_(2,54)_ = 13.84, *p* < 0.0001; post-hoc confirmed a significant difference, *p* = 0.0005, between the 15 mm cylinder and the two smaller ones), and duration of deceleration interval (F_(2,54)_ = 4.92, *p* = 0.011; post-hoc confirmed a significant difference, *p* = 0.003, between the 5 and 15 mm cylinders).

There was a significant viewing condition by distance interaction for peak velocity (F_(2,54)_ = 9.10, *p* = 0.0004) and grasp duration (F_(2,54)_ = 35.72, *p* < 0.0001). Peak velocity increased progressively with distance (*p* < 0.0001) during binocular viewing (40 cm: 1030 ± 173 m/s; 42 cm: 1061 ± 186 m/s; 44 cm:1087 ± 185 m/s). During monocular viewing (40 cm: 947 ± 178 m/s; 42 cm: 968 ± 177 m/s; 44 cm: 971 ± 175 m/s), post hoc test showed significance when comparing the 40 vs. 42 cm distances (*p* = 0.0003) and the 40 cm vs. 44 cm (*p* < 0.0001). During binocular viewing, grasp duration was similar across the three distances (40 cm: 173 ± 48 ms; 42 cm: 175 ± 49 ms; 44 cm:178 ± 49 ms). In contrast, during monocular viewing, grasp duration increased significantly as the distance increased (40 cm: 328 ± 113 ms; 42 cm: 350 ± 123 ms; 44 cm: 395 ± 133 ms, all *p* < 0.0001).

### 3.5. Slope and Intercept Analysis for Reach Peak Velocity

**Figure 6:**
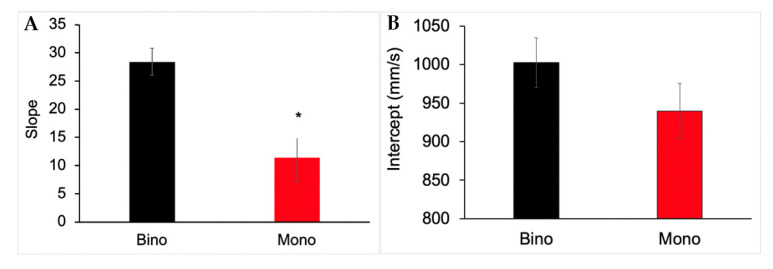
Mean slope (**A**) and intercept (**B**) values from a regression analysis relating peak velocity with distance for reaching during binocular and monocular viewing conditions. Slope was significantly higher during binocular viewing (* *p* < 0.05). Error bars show ±1 standard error.

The mean slope and intercept values for the regression relating peak velocity and distance for binocular and monocular viewing are shown in Figure 6. Statistical analysis confirmed a significant effect of viewing condition for slope (F_(1,27)_ = 12.32, *p* = 0.0009) but not for intercept (F_(1,27)_ = 1.69, *p* = 0.199). Slope was lower during monocular compared to binocular viewing, reinforcing the idea that scaling of peak velocity with distance was reduced (Figure 6A). Although not statistically significant, the intercept was also lower during monocular viewing, suggesting an increased safety margin for reaching movements (Figure 6B).

### 3.6. Safety Margin for Reaching and Grasping

**Figure 7:**
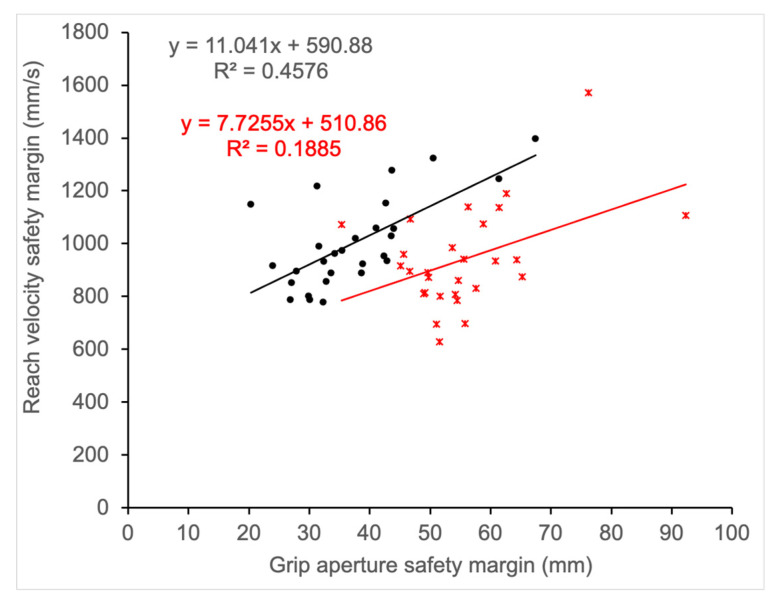
Association between the safety margin for reaching and grasping components of prehension. Individual data points represent the intercept from each participant’s regression analysis relating peak grip aperture and object size (grip aperture safety margin) and reach peak velocity and object distance (reach velocity safety margin). Note that higher safety margin is indicated by higher values on the abscissa and lower values on the ordinate.

The correlation between the reaching and grasping safety margin was significant during binocular (r = 0.68, *p* < 0.0001; 95% CI: 0.41 < r < 0.84) and monocular (r = 0.42, *p* = 0.021; 95% CI: 0.07 < r < 0.69) viewing (Figure 7). The difference in the strength of the correlation did not reach significance (*p* = 0.052). The positive association suggests increased reach peak velocity is associated with a larger grip aperture, suggesting a trade-off relationship between the safety margin for reaching and grasping. In other words, a lower safety margin during reaching (i.e., higher peak velocity) is associated with a higher safety margin for grasping (i.e., larger grip aperture).

## 4. Discussion

This study sought to determine how binocular viewing facilitates size constancy during grasping and manual size estimation. It was expected that size constancy, reflected by grip aperture scaling, would be reduced to a greater extent during monocular viewing for the grasping compared to the estimation task. In contrast to our hypothesis, results showed that size constancy was reduced similarly for both tasks during monocular viewing. Nonetheless, removing binocular cues had a greater impact on grip aperture during grasping compared to estimation. Specifically, monocular viewing was associated with a more variable grip aperture and an increased safety margin during grasping compared to manual estimation. The importance of binocular cues for grasp execution is further highlighted by a one-fold increase in grasp duration during monocular viewing. Overall, these results suggest that removing binocular depth cues impacts grip scaling accuracy similarly for grasping and manual estimation. However, the increased sensory uncertainty regarding object size and/or distance has a greater impact on grasping compared to manual estimation—this was mainly reflected in increased safety margin.

Results from this study demonstrate that binocular cues are important for maintaining size constancy within the peripersonal space. This finding is consistent with a previous study by Chen et al. which showed reduced size constancy in a severely impoverished environment where participants performed the task in the dark while viewing through a pinhole [23]. Notably, in the current study, grasping and manual estimation tasks were performed in a fully lit room where other monocular cues such as relative height, texture gradient, blur, and accommodation were not constrained. Our results indicate that these monocular cues are not sufficient to fully support size constancy, and that binocular depth cues contribute to more accurate grip scaling. The current findings are at odds with a classic study by Leibowitz and Dato [59], which reported no difference in size constancy between binocular and monocular viewing using a perceptual matching task. However, that study examined size constancy in the extrapersonal space over distances ranging between 10 and 120 feet. Given this pattern of results, it is plausible that the contribution of binocular depth cues to size constancy varies with distance: binocular viewing facilitates size constancy at nearer distances whereas monocular cues are sufficient at farther distances. Future studies should test this hypothesis directly.

The idea that the contribution of binocular viewing to size constancy depends on distance is consistent with other studies demonstrating that sensory cues integration is based on their reliability [2,60]. Accordingly, as the utility of stereopsis and ocular vergence falls off with distance [31], it is not surprising that these cues may have a more significant role in maintaining size constancy in the near, peripersonal space. Moreover, stereoscopic depth perception is more accurate (i.e., veridical) within a narrow region close to the body where such information is crucial for handling and manipulating objects [61]. Because accurate scaling of disparity requires distance information, vergence and proprioceptive information from the limb may serve to calibrate and fine tune depth perception [62]. The distinct contribution of stereopsis and ocular vergence cannot be teased apart in the current study as these cues were both present during binocular viewing. Notably, previous work that used prisms and convex lenses to manipulate the vergence demand and stereoacuity reported effects on reach peak velocity and grasping, respectively [33].

In contrast to our hypothesis, the contribution of binocular viewing to size constancy was similar for the grasping and estimation tasks. To our knowledge, this is the first study to demonstrate a comparable reduction in grip aperture scaling during monocular viewing for tasks that engage the action and perception systems. Only one previous study compared grip aperture for a grasping and estimation task during binocular and monocular viewing [43]. That study sought to compare the effect of an illusion on action and perception using a Muller-Lyer figure. Results showed a significant effect of the illusion on the estimation but not the grasping task; however, there was no difference in the average grip aperture between binocular and monocular viewing. The authors did not report the slope relating grip aperture and object size, thus, grip scaling accuracy from that study cannot be compared to the current results. The critical role of binocular viewing for aperture scaling during grasping has been shown in patients with lesions [41,42]; however, it is important to remember that participants in our study were neurologically intact and had normal binocular vision. Thus, activation of disparity-sensitive neurons in the primary visual cortex, as well as dorsal and ventral areas, would be expected during habitual viewing when interacting with objects at different distances. If size constancy is a learned response [63], our results suggest that binocular cues contribute to the development and maintenance of the underlying mechanism for both acting and perceiving.

An interesting finding from our study is the large difference in grip aperture responsiveness between grasping and estimation tasks. Our results for the grasping task are consistent with many previous studies (reviewed by Smeets and Brenner [36]). Similarly, the slope relating grip aperture and object size for the estimation task was within the range of previously reported values [48]. It was previously suggested that absence of visual feedback of the hand during task performance may be a contributing factor and may explain the difference in slope values. Our experiment did not restrict visual feedback during the task; thus, open vs. closed visual feedback cannot fully explain the difference in responsiveness between the tasks. The underlying cause to explain slope differences remains to be established in future studies.

The effect of monocular viewing on prehension has been studied extensively across different tasks [6,7,26,29,64,65]. By and large, most studies demonstrate the benefits of binocular vision for prehension [24,27,66]. Although the improvement during binocular viewing varies across experimental protocols, maximum grip aperture is generally higher and reaching is performed slower during monocular viewing. Our results are aligned with this body of literature: average grip aperture was larger during monocular compared to binocular viewing with a Cohen’s effect size of 1.6, which is comparable to previous studies. In contrast to grasping, not all studies found significant differences between viewing conditions for reach peak velocity [34,67]. Our results showed that peak velocity was reduced on average by 100 mm/s; furthermore, the scaling of peak velocity with reach distance was reduced during monocular viewing. Overall, this pattern of results indicates that participants adopted a larger safety margin for both reaching and grasping when binocular cues were absent.

The safety margin most likely varies with the context of the experiment and examining this variable could help to explain the inconsistent effect of viewing condition on reaching and grasping reported in previous studies. Adopting an appropriate safety margin for task performance is a key feature of human behaviour, which may be learned based on experience [49]. Studies show that participants adopt a larger safety margin when faced with increased sensory uncertainty such as reduced cues [51,52,68], and/or higher motor variability which increases when movements are performed faster [50,53,69]. Here we propose that the safety margin for reaching and grasping can be quantified as the intercept from the regression relating grip aperture and object size, and peak velocity and object distance, respectively. Overall, our results indicate that monocular viewing is associated with an increase in the safety margin for both grasping and reaching, albeit the effect may be greater for grasping because the difference in intercept value between viewing conditions was significant for grasping but did not reach significance for reaching. Comparing the safety margin for grasping and reaching revealed a significant positive association (i.e., higher peak velocity was associated with higher grip aperture), suggesting a trade-off in the safety margins for reaching and grasping. In other words, the potential cost associated with moving the limb faster increases the risk of colliding with the object, which is compensated by adopting a wider grip aperture to ensure that the grasp is successful. Conversely, if the reach is executed more slowly, there is less risk of colliding with the object and the aperture is smaller. Our results are consistent with a study by Wing et al. [53], which showed a significant correlation between reach peak velocity and maximum grip aperture. The current study replicates these results and extends this research by comparing performance during monocular and binocular viewing. Although not statistically significant, the correlation plot reveals that a lower correlation during monocular viewing is due to a larger increase in the safety margin for grasping as compared to reaching. Thus, the absence of binocular vision increases the sensory uncertainty which has a greater impact on grasp programming and grasp application when compared to reach execution. Altogether, our results reinforce the findings from previous studies which clearly demonstrate the importance of binocular depth cues for efficient grasp execution.

It is important to acknowledge the key assumption underlying this investigation and the outcome measure used to test the hypothesis. Specifically, we assumed that object size is used to program maximum grip aperture during prehension. An alternative hypothesis proposes that grip aperture is an emergent characteristic as the digits are moved towards the object [45]. In other words, grip aperture is a by-product of two independently controlled trajectories. Our study was not designed to test this latter hypothesis, however, compelling evidence supporting this view is accumulating (see review by Smeets and colleagues [70]). Results from the current study are not inconsistent with the double pointing hypothesis. Specifically, removing important cues about distance and depth would increase the uncertainty about the object’s location, and thereby influence the digits’ trajectory. One caveat to consider here is that single digit pointing is not associated with a significant change in digit trajectory after peak velocity [71] (i.e., at the time when the maximum grip aperture is measured) or in endpoint precision [65,72]. It is possible that these effects could emerge for tasks that specifically assess two digit pointing.

In conclusion, results from this study demonstrate that binocular viewing facilitates grip aperture scaling similarly for both prehension and manual estimation tasks, suggesting that binocular depth cues contribute to maintaining size constancy. However, the relatively small disruption in aperture scaling during monocular viewing does not explain the large decrement in grasping performance during prehension. In agreement with previous work [73], our results emphasize that removing binocular cues increases the sensory uncertainty impacting both the reach and grasp component which manifest as increased safety margin. A novel contribution stemming from the current work is the trade-off relationship between the safety margin for reaching and grasping.

## Data Availability

All data are available upon request.
